# Bioinformatics Analysis of the Mechanisms of Diabetic Nephropathy *via* Novel Biomarkers and Competing Endogenous RNA Network

**DOI:** 10.3389/fendo.2022.934022

**Published:** 2022-07-14

**Authors:** Mingfei Guo, Yaji Dai, Lei Jiang, Jiarong Gao

**Affiliations:** ^1^ Department of Pharmacy, The Fourth Affiliated Hospital of Anhui Medical University, Hefei, China; ^2^ Department of Pharmacy, Anhui No.2 Provincial People’s Hospital, Hefei, China; ^3^ Department of Pharmacy, The First Affiliated Hospital of Anhui University of Chinese Medicine, Hefei, China

**Keywords:** diabetic neuropathy, bioinformatics analysis, biomarkers, mechanisms, ceRNA

## Abstract

Diabetic nephropathy (DN) is one of the common chronic complications of diabetes with unclear molecular mechanisms, which is associated with end-stage renal disease (ESRD) and chronic kidney disease (CKD). Our study intended to construct a competing endogenous RNA (ceRNA) network *via* bioinformatics analysis to determine the potential molecular mechanisms of DN pathogenesis. The microarray datasets (GSE30122 and GSE30529) were downloaded from the Gene Expression Omnibus database to find differentially expressed genes (DEGs). GSE51674 and GSE155188 datasets were used to identified the differentially expressed microRNAs (miRNAs) and long non-coding RNAs (lncRNAs), respectively. The DEGs between normal and DN renal tissues were performed using the Linear Models for Microarray (limma) package. Gene Ontology (GO) and Kyoto Encyclopedia of Genes and Genomes (KEGG) pathway enrichment analyses were performed to reveal the mechanisms of DEGs in the progression of DN. The protein–protein interactions (PPI) of DEGs were carried out by STRING database. The lncRNA–miRNA–messenger RNA (mRNA) ceRNA network was constructed and visualized *via* Cytoscape on the basis of the interaction generated through the miRDB and TargetScan databases. A total of 94 significantly upregulated and 14 downregulated mRNAs, 31 upregulated and 121 downregulated miRNAs, and nine upregulated and 81 downregulated lncRNAs were identified. GO and KEGG pathways enriched in several functions and expression pathways, such as inflammatory response, immune response, identical protein binding, nuclear factor kappa b (NF-κB) signaling pathway, and PI3K-Akt signaling pathway. Based on the analysis of the ceRNA network, five differentially expressed lncRNAs (DElncRNAs) (SNHG6, KCNMB2-AS1, LINC00520, DANCR, and PCAT6), five DEmiRNAs (miR-130b-5p, miR-326, miR-374a-3p, miR-577, and miR-944), and five DEmRNAs (PTPRC, CD53, IRF8, IL10RA, and LAPTM5) were demonstrated to be related to the pathogenesis of DN. The hub genes were validated by using receiver operating characteristic curve (ROC) and real-time PCR (RT-PCR). Our research identified hub genes related to the potential mechanism of DN and provided new lncRNA–miRNA–mRNA ceRNA network that contributed to diagnostic and potential therapeutic targets for DN.

## Introduction

Diabetic nephropathy (DN) is the most common and serious microvascular complication of diabetes mellitus (DM); the main pathological features are characterized by hyperglycemia, hypertension, and hyperlipidemia (1, 2). Along with the increasing incidence of diabetes, approximately 60%–70% of patients with diabetes developed DN in recent years. Due to lack of thorough and effective treatment, DN is becoming the major cause of renal dysfunction and leads to end-stage of renal disease (ESRD) (3). Despite the many factors that may induce DN, such as obesity, genetics, and environment, the overall molecular mechanisms are still vague. Thus, investigating the mechanisms of DN is prominent in identifying and screening diagnostic biomarkers of the occurrence and development of DN.

Non-coding RNA (ncRNA) is commonly employed for RNAs that do not encode protein, including microRNA (miRNA), long non-coding RNA (lncRNA), circular RNA (circRNA), and PIWI-interacting RNA (piRNA), which are considered as key regulators of gene expression in various diseases and systems (4, 5). As vital players in diverse aspects of biological functions, ncRNAs work with other biomolecules, containing gene activation and silencing, RNA splicing, modification and editing, and protein translation. Over the past decade, RNA-sequencing technologies and comprehensive bioinformatics methods have been greatly promoted, which contributes to the revelation of their essential roles in diabetes and its metabolic syndrome, especially in DN.

**Figure 1:**
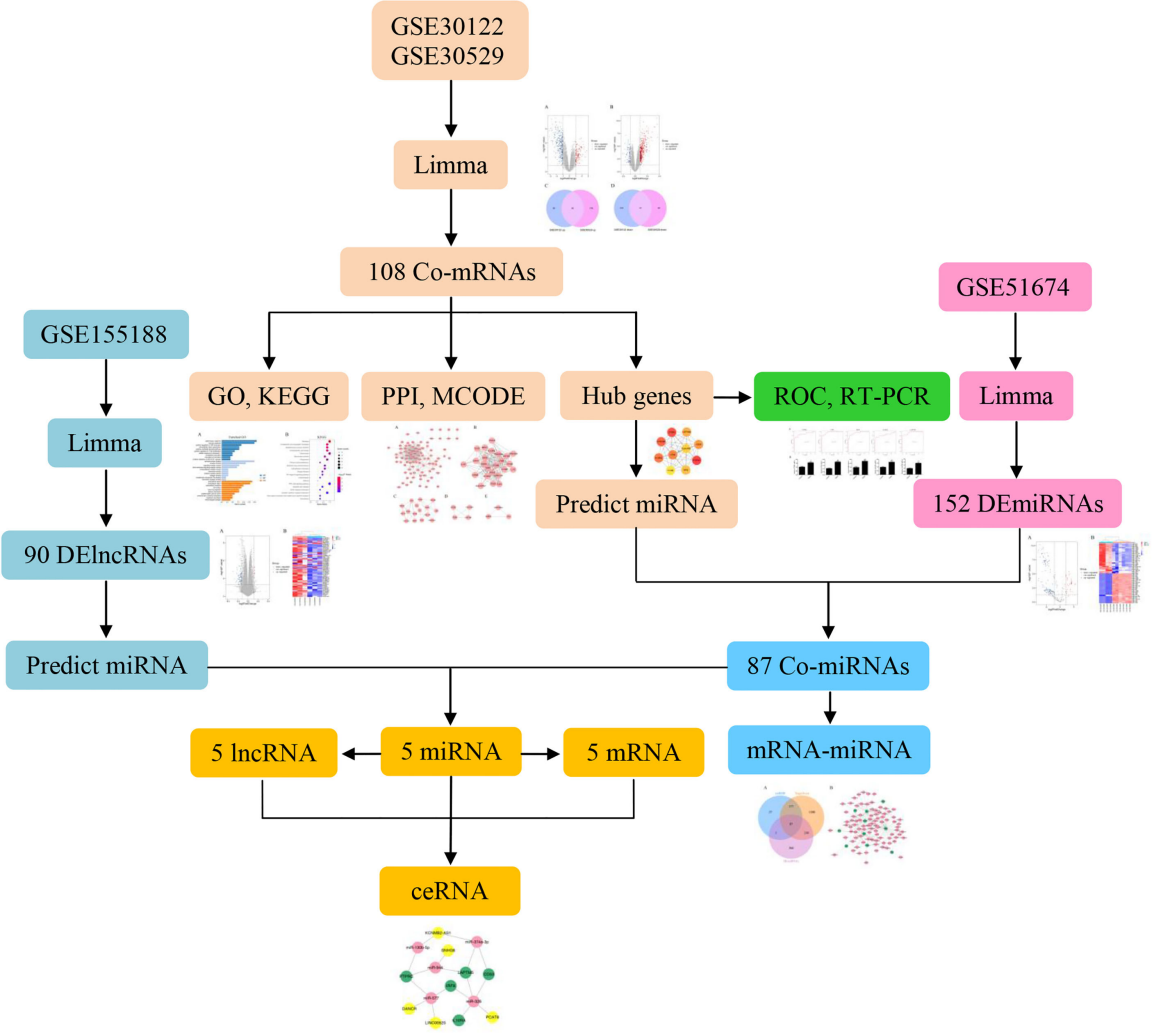
Research flow chart.

Significantly, there is an increasing body of evidence indicating that ncRNAs can act as competing endogenous RNAs (ceRNAs) and play a critical role in the molecular mechanisms of DN. Previous studies have demonstrated that NEAT1/miR-93-5p/CXCL8 and LINC00960/miR-1237-3p/MMP-2 might be potential RNA regulatory pathways to regulate the disease progression of DN (6, 7). Further investigations have illustrated that circ_0037128/miR-17-3p/AKT3 axis significantly negatively regulated cell proliferation and fibrosis in DN pathogenesis (8). Although there is an increasing number of studies showing that lncRNAs in the ceRNA regulate target mRNA expression by competing for shared miRNAs to alleviate the DN, the specific molecular mechanism remains to be poorly understood. Accordingly, the aim of this study was to construct ceRNA networks to identify novel biomarkers and pathogenesis by bioinformatics analysis that will serve as potential diagnosis and therapeutic target for DN. The flow chart is shown in **Figure 1**.

## Materials and Methods

### Microarray Data Collection

The microarray data of the mRNA, miRNA, and lncRNA expression profiles associated with DN were obtained from the Gene Expression Omnibus (GEO, https://www.ncbi.nlm.nih.gov/geo/) database of National Center for Biotechnology Information (NCBI) (9). The following screening criteria were used: “diabetic nephropathy,” “*Homo sapiens*,” and “expression profiling by array.” Eventually, mRNA datasets GSE30122 and GSE30529 met the requirement of the above conditions. In addition, miRNA dataset GSE51674 and lncRNA dataset GSE155188 were identified for further research. Based on all the above datasets, a total of 26 model (DN) and 34 control samples were screened in this research (**Table 1**). The samples were collected from multiple platforms, including Affymetrix Human Genome U133A 2.0 Array, Agilent-029297 Human miRNA Microarray v14 Rev.2, and Agilent-045997 Arraystar human lncRNA microarray V3.

**Table 1:** The microarray dataset information.

Dataset ID	Platform	Model		Control	
		Type	Number	Type	Number
GSE30122	GPL571	Glomeruli	9	Glomeruli	13
GSE30529	GPL571	Tubuli	10	Tubuli	12
GSE51674	GPL10656	Kidney biopsies	4	Kidney biopsies	6
GSE155188	GPL16956	Podocytes	3	Podocytes	3

### Identification of DEGs

The DEGs in the control and DN renal tissues were screened using the limma package in the R software (version 4.0.5), with |log2FC| >1 and p < 0.05 considered significant. Subsequently, the overlap DEGs among the mRNA microarray datasets GSE30122 and GSE30529 were visualized using Venn diagram *via* ImageGP online platform (www.ehbio.com/Cloud_Platform/front/). Additionally, the volcano map was visualized by the R software, which displayed the differential expression data.

### Enrichment Analysis of DEGs

The Gene Ontology (GO) database is a comprehensive and widely used database, which stores extensive information of gene sets including biological process (BP), molecular function (MF), and cell component (CC), and the annotations of genes (10). Kyoto Encyclopedia of Genes and Genomes (KEGG) is an encyclopedia of genomes, which provides abundant genomic information and enriched biological pathways (11). The GO functional annotation and KEGG pathway enrichment involved in DEGs were performed through the online tool DAVID (https://david.ncifcrf.gov/home.jsp). p < 0.05 and gene count ≥2 were regarded as a screening threshold.

### PPI Network Construction and Module Analysis

The PPI network of overlapping DEGs was established based on the Search Tool for the Retrieval of Interacting Genes (STRING, http://string-db.org) database to retrieve the functional interactions among the expressed genes (12). A combined score >0.4 was considered as having a significant interaction. Meanwhile, the analyzed results were imported into the Cytoscape software for integration and visualization (13). The gene clusters were identified *via* MCODE plugin, with degree cutoff = 2, node score cutoff = 0.2, and k-core=2 as filter criteria (14).

### Prediction of Target miRNAs

The differentially expressed miRNAs (DEmiRNAs) were identified from the GSE51674 dataset, with |log2FC| >1 and p < 0.05 as the cutoff criteria. Meanwhile, the volcano plot and heatmap were established to visualize the DEmiRNAs by the R package. Additionally, the downstream target gene prediction was performed by the TargetScan and miRDB databases, and then, the intersection with the DEmiRNAs were taken to obtain candidate miRNAs (15, 16). The mRNA–miRNA network was constructed through the relationship between mRNAs and miRNAs and visualized *via* the Cytoscape.

### Identification of Hub DEGs

The CytoHubba plugin was installed in the Cytoscape software, which was used to screen hub DEGs in the PPI network (17). The hubba values of each DEGs were calculated by the topological algorithms in the CytoHubba plugin. The top 10 genes were selected as hub DEGs.

### Construction of ceRNA Networks

The GSE155188 dataset with |log2FC| >1.5 and p < 0.05 was used to identify the differentially expressed lncRNAs (DElncRNAs). The online database LnaACTdb (http://bio-bigdata.hrbmu.edu.cn/LncACTdb/index.html) and LncTarD (http://bio-bigdata.hrbmu.edu.cn/LncTarD/index.jsp) were used to predict the interactions between lncRNAs and miRNAs. These obtained miRNAs were intersected with the above candidate miRNAs to acquire the final miRNAs. Furthermore, the lncRNAs and mRNAs corresponding to the final miRNAs were selected, respectively. The ceRNA network was constructed by Cytoscape based on the interactions among them.

### Cell Culture

The podocytes of mouse were purchased from Yubo Biotechnology (Shanghai, China) and cultured in Roswell Park Memorial Institute (RPMI) 1640 medium (Hyclone, Logan, UT, USA) containing 5.5 mM glucose, 10% fetal bovine serum (FBS; Thermo Scientific), 100 U/ml penicillin, and 100 µg/ml streptomycin (Solarbio, Beijing, China) at 37°C, 95% humidity, and 5% CO_2_. Controls were cultured in normal medium containing 5.5 mM glucose. Meanwhile, the cells were pretreated with 30 mM high glucose (HG) for 24 h to establish HG-induced podocyte model.

### RT-PCR Validation of Hub Genes

Total RNA was isolated by TRIzol reagent (Thermo Fisher Scientific) in accordance with the manufacturer’s instructions. The isolated RNA was reverse transcribed into cDNA by M-MuLV First-Strand cDNA Synthesis Kit (Sangon Biotech, Shanghai, China). Subsequently, quantitative real-time PCR was performed using the iTaq Universal SYBR Green Supermix kit (Bio-Rad, USA) and an ABI 7500 PCR system (ABI, USA). The relative expression of hub genes was normalized to glyceraldehyde 3-phosphate dehydrogenase (GAPDH) and determined using the 2^−ΔΔCt^ method. The primer sequences are listed in **Supplementary Table S1**.

### Statistics Analysis

The SPSS 20.0 was used for statistical analysis. GraphPad Prism 8 and R software (version 4.0.5) were used to construct statistical graphs. The Student’s t-test was conducted to analyze the differences between the two groups. A p-value of <0.05 was considered as statistically significant.

## Results

### Identification of DEGs

**Figure 2:**
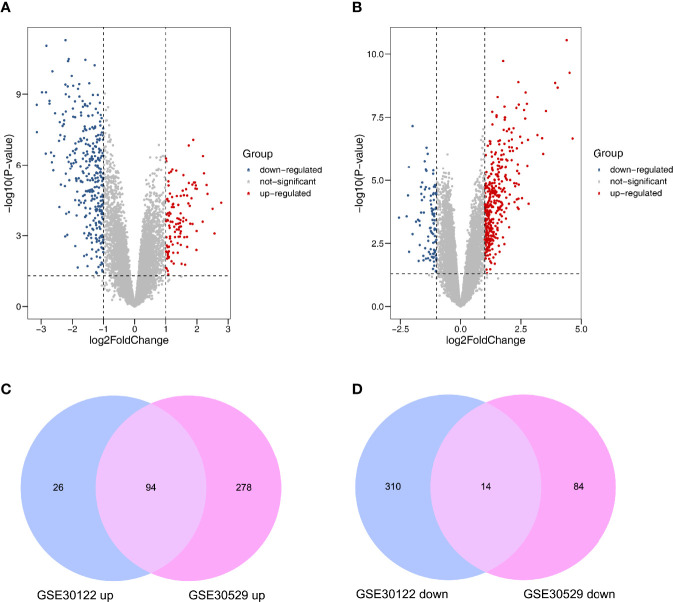
Differently expressed mRNAs in DN. **(A)** GSE30122. **(B)** GSE30529. Red and blue dots represent up- and downregulated genes, and gray dots represent no differential expression genes. **(C)** Coexpression of upregulated genes. **(D)** Coexpression of downregulated genes.

After normalizing the microarray datasets, we identified 444 DEGs in GSE30122. It contained 120 upregulated and 324 downregulated genes (**Supplementary Table S2**). Similarly, in GSE30529, 470 DEGs were screened, of which 372 were upregulated and 98 were downregulated genes (**Supplementary Table S3**). The DEmRNAs between the two groups were shown in volcano plots (**Figures 2A, B**). After intersecting the two datasets, 94 upregulated and 14 downregulated mRNAs were screened out. The analysis results were visualized by a Venn diagram (**Figures 2C, D**).

### GO and KEGG Pathway Analysis

**Figure 3:**
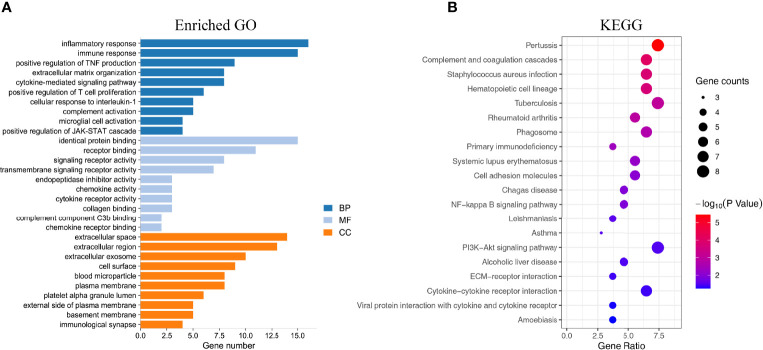
Enrichment analysis of the DEGs. **(A)** The top 10 enriched GO analysis results. **(B)** The top 20 KEGG pathways results. The size of the spots indicates the number of genes, and the color indicates the p-value.

GO function and KEGG pathway enrichment analyses of DEGs were performed by the DAVID online software. GO enrichment analysis was performed on the DEGs functions, consisting of biological process (BP), molecular function (MF), and cell component (CC). In the GO enrichment results, the DEGs in BP were mainly enriched in inflammatory response, immune response, complement activation, positive regulation of T-cell proliferation, extracellular matrix organization, etc. The MF analysis revealed that the DEGs were significantly involved in identical protein binding, receptor binding, signaling receptor activity, transmembrane signaling receptor activity, chemokine activity, etc. As for CC, the DEGs were mainly focused on extracellular region, extracellular space, cell surface, blood microparticle, plasma membrane, basement membrane, etc. The top 10 remarkably enriched GO terms are listed in **Figure 3A**. Additionally, KEGG pathway analysis results were displayed in complement and coagulation cascades, NF-κB signaling pathway, PI3K-Akt signaling pathway, ECM–receptor interaction, primary immunodeficiency, cytokine–cytokine receptor interaction, etc. The top 20 KEGG pathways are shown in **Figure 3B**.

### PPI Network and Gene Cluster Analysis

**Figure 4:**
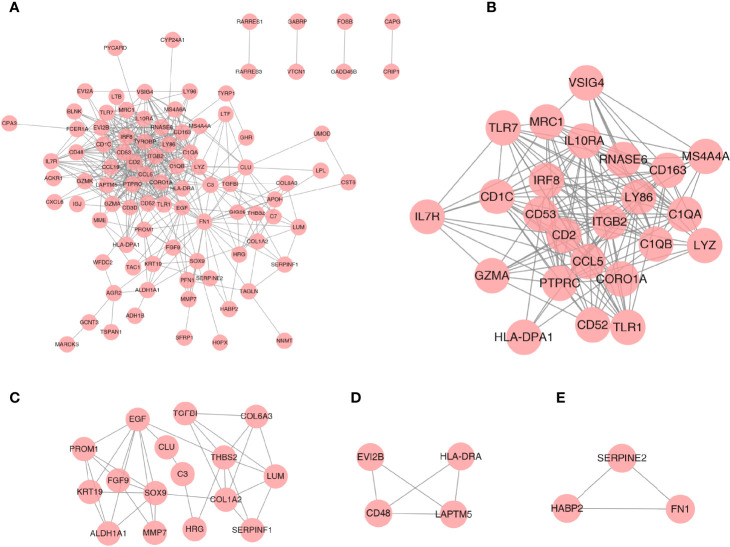
PPI network analysis and gene cluster identification. **(A)** PPI network of DEGs. **(B–E)** Four modules extracted from the PPI network. The node represents the genes; the edge represents the relationship between them.

A PPI network was established based on the DEGs with a combined score >0.4 for visualization by Cytoscape. A total of 93 DEGs from 108 coexpressed DEGs were contained into the network, which contained 93 nodes and 435 edges (**Figure 4A**). Furthermore, 15 DEGs were discarded because they did not interact with others. In addition, four gene clusters were distinguished through the MCODE analysis (**Figures 4B–E**); the scores were 12.870, 4.667, 3.333, and 3.000, respectively.

### Hub Gene Identification

**Figure 5:**
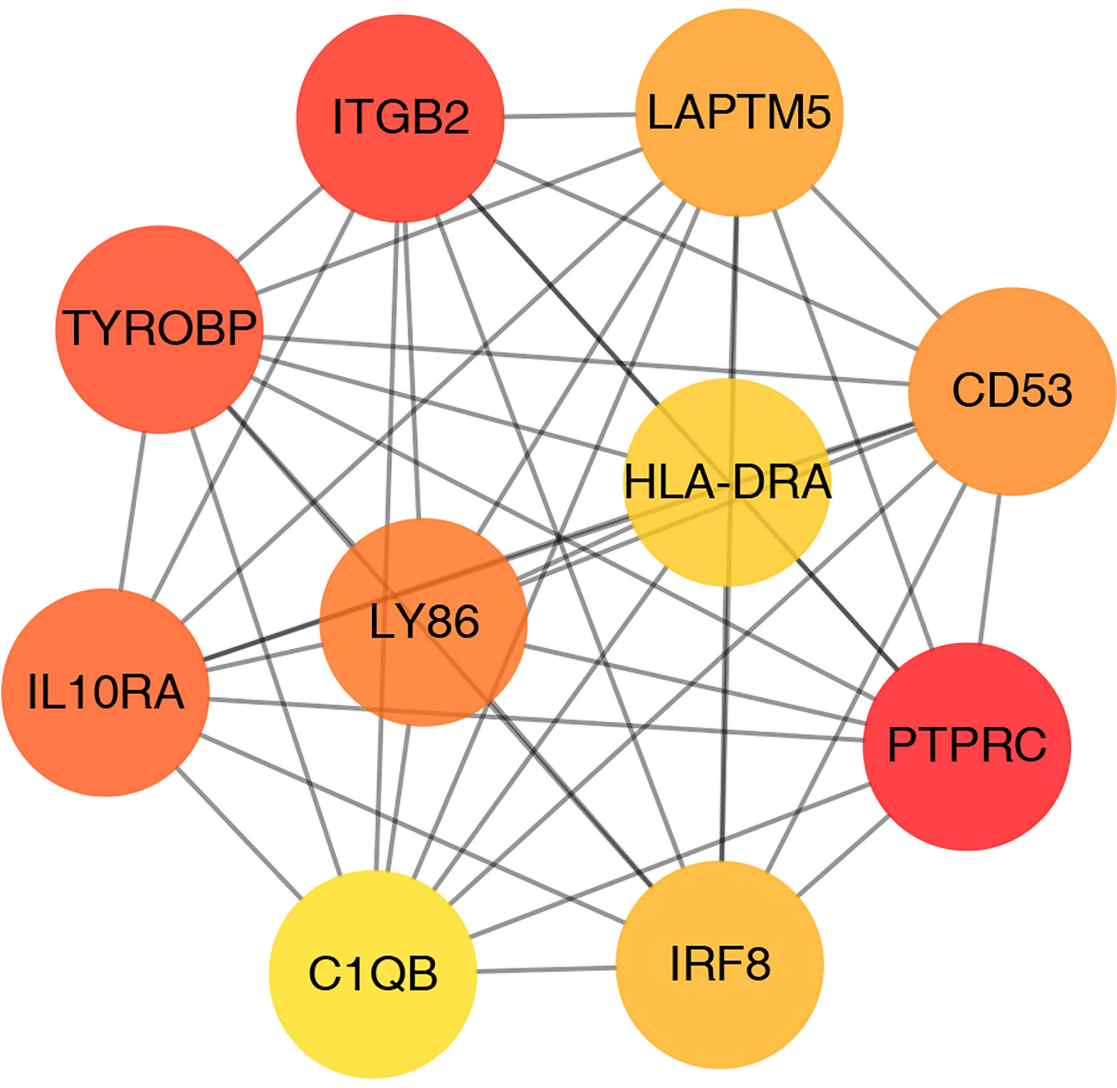
Screening of hub genes.

CytoHubba plugin was applied to explore the hub genes in the PPI network. The top 10 hub genes were identified by intersecting the results from the 11 algorithms of CytoHubba including Closeness, EcCentricity, Radiality, BottleNeck, Stress Betweenness, and Degree (**Figure 5**). Among them, a detailed information of these hub genes is shown in **Table 2**.

**Table 2:** The top 10 hub genes identified and their information.

Gene	Description	Degree	BetweennessCentrality
PTPRC	protein tyrosine phosphatase, receptor type C	40	0.2161
ITGB2	Integrin beta-2	30	0.0557
TYROBP	TYRO protein tyrosine kinase-binding protein	29	0.0444
IL10RA	Interleukin-10 receptor subunit alpha	27	0.0246
LY86	Lymphocyte antigen 86	25	0.0164
C1QB	Complement C1q subcomponent subunit B	23	0.0324
CD53	Leukocyte surface antigen CD53	22	0.0198
IRF8	Interferon regulatory factor 8	21	0.0062
LAPTM5	Lysosomal-associated transmembrane protein 5	18	0.0121
HLA-DRA	HLA class II histocompatibility antigen, DR alpha chain	18	0.0037

### Identification of Differentially Expressed miRNAs

**Figure 6:**
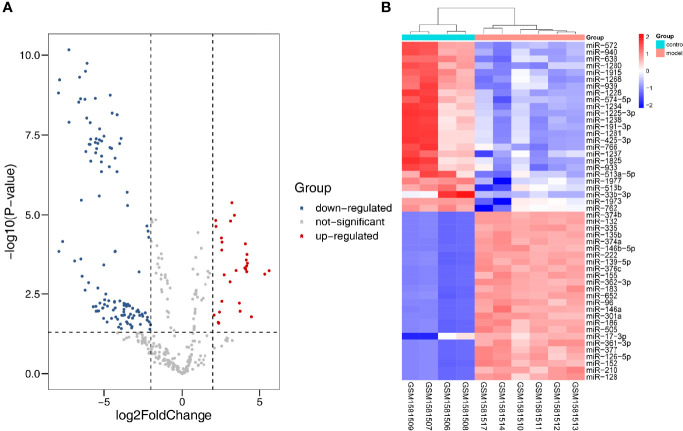
Differentially expressed miRNA in DN. **(A)** Red dots indicate upregulation, blue dots indicate downregulation, and gray dots indicate no differential expression. **(B)** Red represents upregulation, and blue represents downregulation.

In all, 10 samples and 887 miRNAs from the GSE51674 dataset were identified. Volcano plots were plotted through the R package, with |log2FC| > 2 and p < 0.05. Finally, 31 upregulated and 121 downregulated DEmiRNAs were screened out (**Figure 6A**). The top 50 differential genes were selected, and a heatmap was constructed to visualize the differential expression data (**Figures 6B**).

### Construction of mRNA–miRNA Network

**Figure 7:**
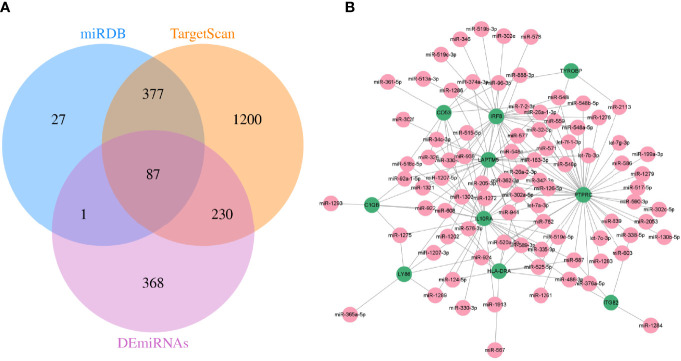
The mRNA–miRNA network was constructed. **(A)** Screening of co-expressed miRNAs. **(B)** miRNA–mRNA regulatory network of hub genes. Green and red indicate mRNA and miRNA, respectively.

Meanwhile, TargetScan and miRDB databases were utilized to further predict the target miRNAs of hub genes. Then, the intersection with the DEmiRNAs was taken by Venn diagrams (**Figure 7A**). Subsequently, a total of 87 miRNAs were detected, and a visualized mRNA–miRNA network was established by Cytoscape (**Figure 7B**).

### Identification of Differentially Expressed LncRNAs

**Figure 8:**
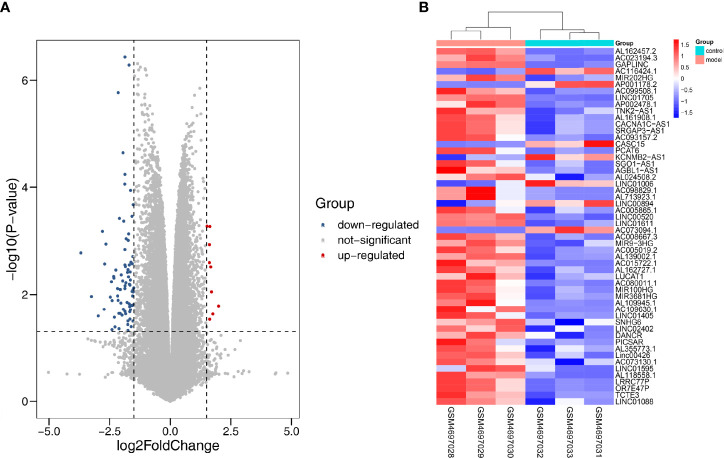
Differentially expressed lncRNA in DN. **(A)** Red dots represent upregulation, blue dots represent downregulation, and gray dots represent no differential expression. **(B)** Red represents upregulation, and blue represents downregulation.

We applied |log2FC| > 1.5, p < 0.05 as the standard to analyze lncRNA dataset from the GSE155188. Subsequently, 93 DElncRNAs were identified, including nine upregulated and 81 downregulated genes. The “pheatmap” and “ggplot2” R packages were used to draw heatmap and volcanos of the DElncRNAs (**Figures 8A, B**).

### Construction of ceRNA Network

**Figure 9:**
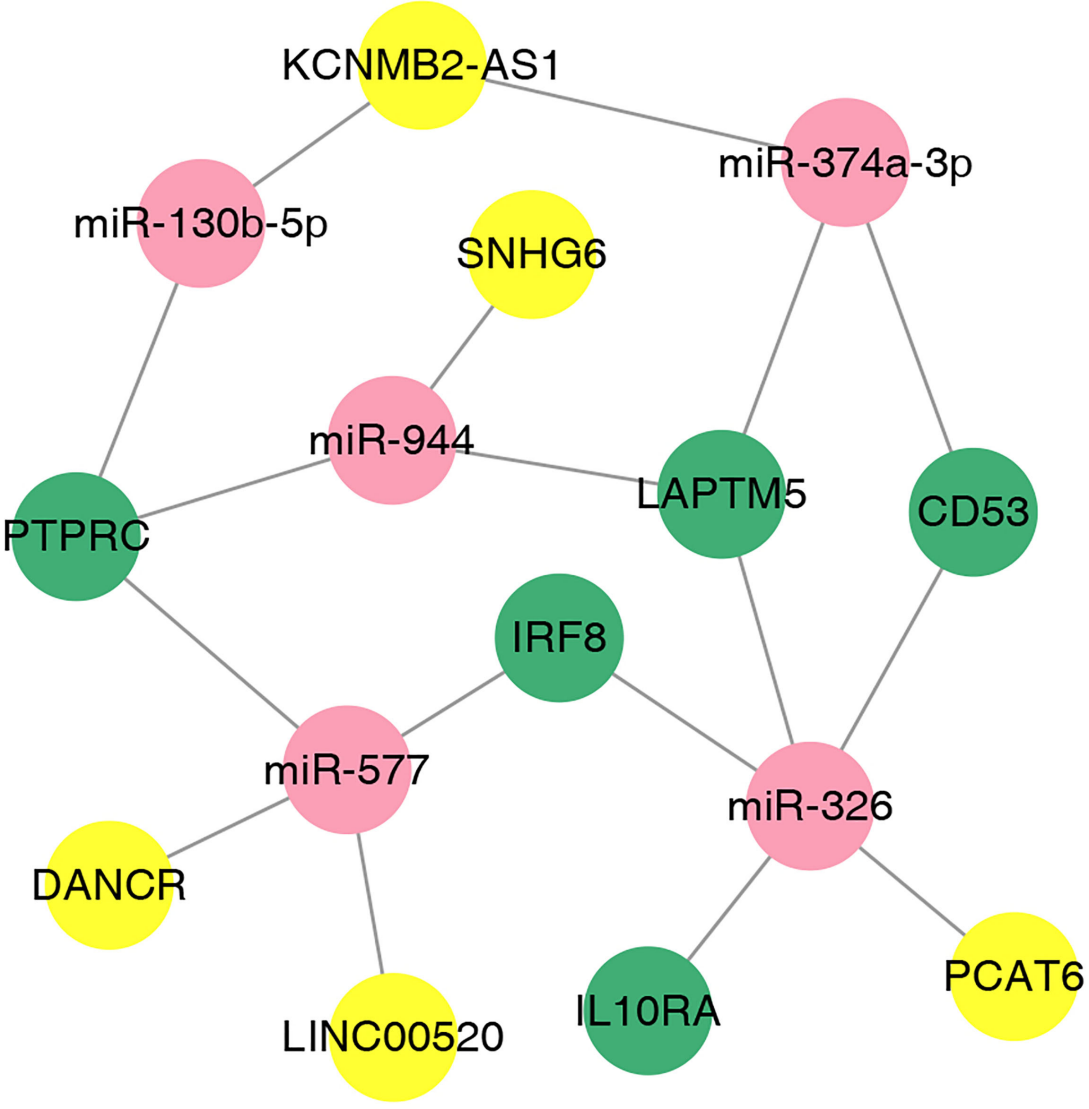
The lncRNA–miRNA–mRNA ceRNA network in DN. The nodes colored in yellow, red, and green represent lncRNAs, miRNAs, and mRNAs, respectively.

We used the online database LnaACTdb and LncTarD to predict the miRNAs that interact with the selected lncRNAs. Meanwhile, 17 lncRNAs were screened from the 90 DElncRNAs (**Table 3**), corresponding to 74 miRNAs. Eventually, 74 miRNAs were intersected with the above 87 coexpressed miRNAs; then, five miRNAs were selected, and five corresponding mRNAs were identified. Subsequently, the ceRNA network was constructed and illustrated by Cytoscape. This network contained five lncRNAs, five miRNAs, and five mRNAs (**Figure 9**). According to the ceRNA hypothesis, we will integrate to understand the complex regulatory relationship between the lncRNAs, miRNAs, and mRNAs in DN.

**Table 3:** The 17 lncRNAs identified and their information.

ID	lncRNA	p-value	logFC
ASHGA5P049942	GAPLINC	4.31E−06	−1.14739
ASHGA5P056005	LINC00520	8.42E−06	−1.35597
ASHGA5P032711	LINC01705	8.59E−06	−1.07753
ASHGA5P017664	Linc00426	3.30E−03	−1.00102
ASHGA5P058628	CASC15	6.18E−03	1.117783
ASHGA5P055201	MIR100HG	7.73E−03	−2.17639
ASHGA5P028607	AL139002.1	3.90E−04	−1.19673
ASHGA5P051944	DANCR	1.60E−02	−1.56338
ASHGA5P043444	LINC01006	1.65E−02	1.400726
ASHGA5P037365	PICSAR	2.56E−02	−2.96829
ASHGA5P044351	SNHG6	3.39E−02	−1.08823
ASHGA5P039263	TNK2-AS1	5.86E−03	−1.07908
ASHGA5P015966	PCAT6	8.90E−03	−1.52594
ASHGA5P018300	KCNMB2-AS1	8.91E−03	1.698157
ASHGA5P020436	LUCAT1	1.39E−02	−1.18895
ASHGA5P040002	LINC01088	1.39E−02	−1.31517
ASHGA5P054160	LINC00894	3.17E−02	1.203263

### Validation of Hub Genes

**Figure 10:**
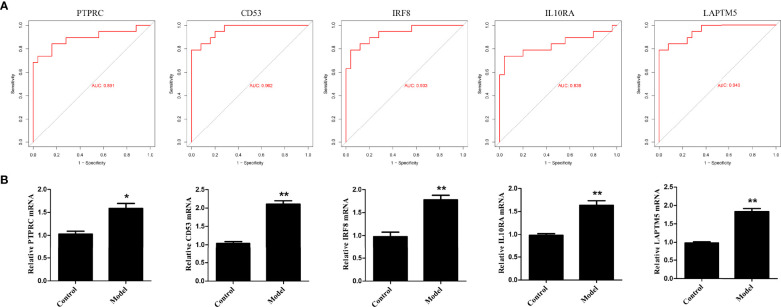
Validation of hub genes. **(A)** ROC curve of the hub genes in GEO datasets. **(B)** The relative expression of hub genes was detected by RT-PCR. ^*^p < 0.05, ^**^p < 0.01.

To validate the bioinformatics analysis results, the receiver operating characteristic curve (ROC) was plotted and the area under the curve (AUC) was calculated to distinguish DN from controls (**Figure 10A**). The diagnostic values of these hub genes are follows: PTPRC (AUC, 0.891), CD53 (AUC, 0.962), IRF8 (AUC, 0.933), IL10RA (AUC, 0.838) and LAPTM5 (AUC, 0.949). In addition, RT-PCR assay was used to investigate the expression of the five hub genes. As shown in **Figure 10B**, the relative expression of PTPRC, CD53, IRF8, IL10RA, and LAPTM5 was also significantly upregulated in the HG-induced podocyte models compared to that in controls.

## Discussion

The prevalence of T2DM has been growing dramatically worldwide in recent years. DN is one of the serious complications of T2DM, which has become the primary cause of ESRD (18). The glomerular hypertrophy and glomerular and tubular basement membrane thickening played a vital role in the pathogenesis of DN (19, 20). Nevertheless, owing to the complexity of its etiology, the molecular mechanisms of DN is still poorly understood. Hence, it is imperative to explore the new potential biomarkers for the treatment and diagnosis of DN.

In this study, the comprehensive bioinformatics methods were adopted to identify differentially expressed genes in DN and control samples. A total of 108 DEGs were screened, of which 94 were upregulated and 14 were downregulated genes. GO analysis revealed that the biological functions of DEGs were mainly involved in identical protein binding, receptor binding, signaling receptor activity, transmembrane signaling receptor activity, and chemokine activity. The KEGG pathway analysis results were significantly enriched in complement and coagulation cascades, NF-κB signaling pathway, PI3K-Akt signaling pathway, and ECM–receptor interaction. It was also revealed that immune and inflammatory responses were also involved in the occurrence and development of DN. In addition, CytoHubba plugin analysis revealed that 10 hub genes were identified from the PPI network. Further analysis demonstrated that PTPRC, CD53, IRF8, IL10RA, and LAPTM5 perhaps have the essential functions in molecular mechanisms of DN.

PTPRC, also known as CD45, expressed on nucleated cells of the hematopoietic system, is an essential regulator of B- and T-cell receptor signaling (21, 22). In recent years, many studies have reported that CD45 had promoted the development of diabetes and its metabolic syndrome. Studies have confirmed that the expression of PTPRC was related to β-cell function in DM, which directly influenced insulin secretion and glycemic control (23). Our results indicated that a higher expression of PTPRC was closely related to DN. Therefore, we speculated that the enhancement of PTPRC expression caused the impairment of insulin secretion and sensitivity, thus aggravating the DN symptom.

CD53 is a member of the tetraspanin family that regulates key processes in immune cells (24, 25). In particular, it plays an essential role in cytokine regulation and interaction between natural killer cells and antigen-presenting cells (26). However, its contribution in the setting of DN has not been fully elucidated. Indeed, prior studies have indicated that inflammation and immune cell infiltration are increasingly recognized as central to the disease progression in DN patients (27, 28). In this study, we showed that CD53 expression is minimal in control samples, in which the lack in CD53 has significantly decreased the risk of hyperglycemia, proteinuria, and inflammatory response.

IRF8, as an interferon (IFN) regulatory factor, is crucial in the development of dendritic cells. The research indicated that dendritic cells could promote the presentation of autoantigen and activation of diabetogenic T cells, which plays vital roles in insulin secretion and regulation of hyperglycemia function (29). In diabetic mice, inhibited microglial activation and inflammatory response will regulate the expressions of IRF8, eventually leading to the improvement of diabetic retinopathy (30). Actually, diabetic patients with low IRF8 transcriptional activity have lower levels of blood glucose and blood lipid and high insulin content. This is consistent with our study in which we found that IRF8 expression was overexpressed in DN compared to the control groups.

IL10RA, as receptor of IL10, is also associated with the immune and inflammatory responses (31). Similarly, LAPTM5, a lysosomal membrane protein, is preferentially expressed in immune and hematopoietic cells (32). There are powerful results suggesting that it may play a vital role for the immunological aspects in etiologies of DN. Hyperglycemia easily triggers an inflammatory response, which indirectly promotes the expression of IL10RA and aggravates DN. In addition, the latest research demonstrated that LAPTM5, which is increased in obese patients, may be a hub target for obesity treatment (33). Coincidently, we found that LAPTM5 was upregulated in DN samples and downregulated in the control group. Hence, we hypothesized that inactivation of LAPTM5 may contribute to the treatment of diabetes and its complications.

Furthermore, a ceRNA network about target miRNAs and lncRNAs of their mRNAs, namely, PTPRC, CD53, IRF8, IL10RA, and LAPTM5, was constructed based on the interactions. Previous studies have shown that downregulation of miR-130b-5p could significantly reduce atherosclerosis, prevent insulin resistance, and alleviate diabetes symptoms (34, 35). Meanwhile, regulating miR-326 may decrease the expression of HbAc1, which participates in the pathogenesis of DN process (36). The silencing of miR-326 was also demonstrated to induce proliferation and reduce apoptosis in high-glucose-induced renal tubular epithelial cells in DN (37). Moreover, miR-374a-3p may regulate acute kidney injury by targeting TLR4/NF-κB pathway to reduce the rate of apoptosis and the generation of inflammatory cytokines (38). Most studies suggested that miR-577 and miR-944 are more commonly expressed in cancers, such as ovarian cancer (39), gastric cancer (40), and colorectal cancer (41), and little is known regarding in T2DM. Nevertheless, recent studies have shown that these genes also participate in fasting glucose control in diabetes *via* activating Akt signaling pathways and increasing insulin secretion (42, 43).

In addition, lncRNA SNHG6, LINC00520, PCAT6, KCNMB2-AS1, and DANCR, serving as ceRNAs, have been reported to be regulated in multiple cancers. Beyond all expectations, our findings declared a novel underlying molecular mechanism of SNHG6/miR-944, LINC00520/miR-577, PCAT6/miR-326 DANCR/miR-577, and KCNMB2-AS1/miR-374a-3p signaling axis in promoting DN progression, which indicates that these genes may serve as a therapeutic target of DN. However, further experiments are required to validate their effects and mechanisms to support the hypothesis.

Taken together, we identified five hub genes, namely, PTPRC, CD53, IRF8, IL10RA, and LAPTM5, as potential biomarkers for the diagnosis and treatment of DN and revealed the mechanism of DN at the transcriptome level. In addition, we propose that SNHG6-miR-944-PTPRC/LAPTM5, LINC00520/DANCR-miR-577-PTPRC/IRF8, PCAT6-miR-326-LAPTM5/IRF8/IL10RA/CD53, KCNMB2-AS1-miR-374a-3p/LAPTM/CD53, and KCNMB2-AS1-miR-130b-5p-PTPRC are potential ceRNA regulatory pathways that are involved in the regulation of DN.

## Data Availability Statement

The original contributions presented in the study are included in the article/**Supplementary Material**. Further inquiries can be directed to the corresponding author.

## Author Contributions

MFG designed the experiment and wrote the original draft. YJD helped in the acquisition, analysis, and interpretation of the datasets, and wrote the revised manuscript. LJ helped in the datasets analysis. JRG revised this manuscript for important intellectual content. All authors contributed to the article and approved the submitted version.

## Funding

This work is financially supported by the Research Fund of Anhui Medical University (Grant No. 2021xkj056) and the Key Research and Development Plan Projects of Anhui Province (Grant No. 2022e07020024).

## Conflict of Interest

The authors declare that the research was conducted in the absence of any commercial or financial relationships that could be construed as a potential conflict of interest.

## Publisher’s Note

All claims expressed in this article are solely those of the authors and do not necessarily represent those of their affiliated organizations, or those of the publisher, the editors and the reviewers. Any product that may be evaluated in this article, or claim that may be made by its manufacturer, is not guaranteed or endorsed by the publisher.

## References

[1] Samsu N. Diabetic Nephropathy: Challenges in Pathogenesis, Diagnosis, and Treatment. BioMed Res Int (2021) 2021:1497449. doi: 10.1155/2021/1497449 34307650 PMC8285185

[2] Li KX Ji MJ Sun HJ. An Updated Pharmacological Insight of Resveratrol in the Treatment of Diabetic Nephropathy. Gene (2021) 780:145532. doi: 10.1016/j.gene.2021.145532 33631244

[3] Quan KY Yap CG Jahan NK Pillai N. Review of Early Circulating Biomolecules Associated With Diabetes Nephropathy-Ideal Candidates for Early Biomarker Array Test for DN. Diabetes Res Clin Pract (2021) 182:109122. doi: 10.1016/j.diabres.2021.109122 34742785

[4] Lorenzi L Chiu HS Avila Cobos F Gross S Volders PJ Cannoodt R. The RNA Atlas Expands the Catalog of Human Non-Coding RNAs. Nat Biotechnol (2021) 39(11):1453–65. doi: 10.1038/s41587-021-00936-1 34140680

[5] Lu J Huang Y Zhang X Xu Y Nie S. Noncoding RNAs Involved in DNA Methylation and Histone Methylation, and Acetylation in Diabetic Vascular Complications. Pharmacol Res (2021) 170:105520. doi: 10.1016/j.phrs.2021.105520 33639232

[6] Li G Zhang J Liu D Wei Q Wang H Lv Y. Identification of Hub Genes and Potential ceRNA Networks of Diabetic Nephropathy by Weighted Gene Co-Expression Network Analysis. Front Genet (2021) 12:767654. doi: 10.3389/fgene.2021.767654 34790229 PMC8591079

[7] Yu Y Jia YY Wang M Mu L Li HJ. PTGER3 and MMP-2 Play Potential Roles in Diabetic Nephropathy *via* Competing Endogenous RNA Mechanisms. BMC Nephrol (2021) 22(1):27. doi: 10.1186/s12882-020-02194-w 33435900 PMC7805187

[8] Wang Q Cang Z Shen L Peng W Xi L Jiang X. Circ_0037128/miR-17-3p/AKT3 Axis Promotes the Development of Diabetic Nephropathy. Gene (2021) 765:145076. doi: 10.1016/j.gene.2020.145076 32860899

[9] Clough E Barrett T. The Gene Expression Omnibus Database. Methods Mol Biol (2016) 1418:93–110. doi: 10.1007/978-1-4939-3578-9_5 27008011 PMC4944384

[10] The Gene Ontology Consortium. The Gene Ontology Resource: 20 Years and Still GOing Strong. Nucleic Acids Res (2019) 47(D1):D330–8. doi: 10.1093/nar/gky1055 PMC6323945 30395331

[11] Kanehisa M Furumichi M Tanabe M Sato Y Morishima K. KEGG: New Perspectives on Genomes, Pathways, Diseases and Drugs. Nucleic Acids Res (2017) 45(D1):D353–61. doi: 10.1093/nar/gkw1092 PMC5210567 27899662

[12] Zhang L Han L Wang X Wei Y Zheng J Zhao L. Exploring the Mechanisms Underlying the Therapeutic Effect of Salvia Miltiorrhiza in Diabetic Nephropathy Using Network Pharmacology and Molecular Docking. Biosci Rep (2021) 41(6):BSR20203520. doi: 10.1042/BSR20203520 33634308 PMC8209169

[13] Cai W Li H Zhang Y Han G. Identification of Key Biomarkers and Immune Infiltration in the Synovial Tissue of Osteoarthritis by Bioinformatics Analysis. PeerJ (2020) 8:e8390. doi: 10.7717/peerj.8390 31988808 PMC6970550

[14] Nangraj AS Selvaraj G Kaliamurthi S Kaushik AC Cho WC Wei DQ. Integrated PPI-And WGCNA-Retrieval of Hub Gene Signatures Shared Between Barrett’s Esophagus and Esophageal Adenocarcinoma. Front Pharmacol (2020) 11:881. doi: 10.3389/fphar.2020.00881 32903837 PMC7438937

[15] Li H Liang J Wang J Han J Li S Huang K. Mex3a Promotes Oncogenesis Through the RAP1/MAPK Signaling Pathway in Colorectal Cancer and is Inhibited by hsa-miR-6887-3p. Cancer Commun (Lond) (2021) 41(6):472–91. doi: 10.1002/cac2.12149 PMC8211350 33638620

[16] Nooh M Hakemi-Vala M Nowroozi J Fatemi SR Dezfulian M. Prediction of Blood miRNA-mRNA Regulatory Network in Gastric Cancer. Rep Biochem Mol Biol (2021) 10(2):243–56. doi: 10.52547/rbmb.10.2.243 PMC8480291 34604414

[17] Ma H He Z Chen J Zhang X Song P. Identifying of Biomarkers Associated With Gastric Cancer Based on 11 Topological Analysis Methods of CytoHubba. Sci Rep (2021) 11(1):1331. doi: 10.1038/s41598-020-79235-9 33446695 PMC7809423

[18] Liu XQ Jiang L Lei L Nie ZY Zhu W Wang S. Carnosine Alleviates Diabetic Nephropathy by Targeting GNMT, A Key Enzyme Mediating Renal Inflammation and Fibrosis. Clin Sci (Lond) (2020) 134(23):3175–93. doi: 10.1042/CS20201207 PMC7726623 33241846

[19] Lee JY Yang JW Han BG Choi SO Kim JS. Adiponectin for the Treatment of Diabetic Nephropathy. Korean J Intern Med (2019) 34(3):480–91. doi: 10.3904/kjim.2019.109 PMC6506734 31048658

[20] Zhou H Ni WJ Meng XM Tang LQ. MicroRNAs as Regulators of Immune and Inflammatory Responses: Potential Therapeutic Targets in Diabetic Nephropathy. Front Cell Dev Biol (2021) 8:618536. doi: 10.3389/fcell.2020.618536 33569382 PMC7868417

[21] Al Barashdi MA Ali A McMullin MF Mills K. Protein Tyrosine Phosphatase Receptor Type C (PTPRC or CD45). J Clin Pathol (2021) 74(9):548–52. doi: 10.1136/jclinpath-2020-206927 PMC8380896 34039664

[22] Wei J Fang D Zhou W. CCR2 and PTPRC are Regulators of Tumor Microenvironment and Potential Prognostic Biomarkers of Lung Adenocarcinoma. Ann Transl Med (2021) 9(18):1419. doi: 10.21037/atm-21-3301 34733971 PMC8506762

[23] Lopez-Perez D Redruello-Romero A Garcia-Rubio J Arana C Garcia-Escudero LA Tamayo F. In Obese Patients With Type 2 Diabetes, Mast Cells in Omental Adipose Tissue Decrease the Surface Expression of CD45, CD117, CD203c, and FcϵRI. Front Endocrinol (Lausanne) (2022) 13:818388. doi: 10.3389/fendo.2022.818388 35370964 PMC8965342

[24] Dunlock VE. Tetraspanin CD53: An Overlooked Regulator of Immune Cell Function. Med Microbiol Immunol (2020) 209(4):545–52. doi: 10.1007/s00430-020-00677-z PMC7395052 32440787

[25] Demaria MC Yeung L Peeters R Wee JL Mihaljcic M Jones EL. Tetraspanin CD53 Promotes Lymphocyte Recirculation by Stabilizing L-Selectin Surface Expression. iScience (2020) 23(5):101104. doi: 10.1016/j.isci.2020.101104 32428859 PMC7232089

[26] Jin HS Cho JE Park S. Association Between CD53 Genetic Polymorphisms and Tuberculosis Cases. Genes Genomics (2019) 41(4):389–95. doi: 10.1007/s13258-018-0764-3 30506122

[27] Li J Bao L Zha D Zhang L Gao P Zhang J. Oridonin Protects Against the Inflammatory Response in Diabetic Nephropathy by Inhibiting the TLR4/p38-MAPK and TLR4/NF-κb Signaling Pathways. Int Immunopharmacol (2018) 55:9–19. doi: 10.1016/j.intimp.2017.11.040 29207360

[28] Kuo HL Huang CC Lin TY Lin CY. IL-17 and CD40 Ligand Synergistically Stimulate the Chronicity of Diabetic Nephropathy. Nephrol Dial Transplant (2018) 33(2):248–56. doi: 10.1093/ndt/gfw397 28339909

[29] Besin G Gaudreau S Dumont-Blanchette E Ménard M Guindi C Dupuis G. IFN Regulatory Factors 4 and 8 Expression in the NOD Mouse. Clin Dev Immunol (2011) 2011:374859. doi: 10.1155/2011/374859 21647406 PMC3102445

[30] Liu X Xu B Gao S. Spleen Tyrosine Kinase Mediates Microglial Activation in Mice With Diabetic Retinopathy. Transl Vis Sci Technol (2021) 10(4):20. doi: 10.1167/tvst.10.4.20 PMC8083065 34003998

[31] Kapelski P Skibinska M Maciukiewicz M Pawlak J Zaremba D Twarowska-Hauser J. Family-Based Association Study of Interleukin 10 (IL10) and Interleukin 10 Receptor Alpha (IL10RA) Functional Polymorphisms in Schizophrenia in Polish Population. J Neuroimmunol (2016) 297:92–7. doi: 10.1016/j.jneuroim.2016.05.010 27397081

[32] Chen L Wang G Luo Y Wang Y Xie C Jiang W. Downregulation of LAPTM5 Suppresses Cell Proliferation and Viability Inducing Cell Cycle Arrest at G0/G1 Phase of Bladder Cancer Cells. Int J Oncol (2017) 50(1):263–71. doi: 10.3892/ijo.2016.3788 27922670

[33] Yan K Zhang P Jin J Chen X Guan H Li Y. Integrative Analyses of Hub Genes and Their Association With Immune Infiltration in Adipose Tissue, Liver Tissue and Skeletal Muscle of Obese Patients After Bariatric Surgery. Adipocyte (2022) 11(1):190–201. doi: 10.1080/21623945.2022.2060059 35412419 PMC9009953

[34] Coban N Ozuynuk AS Erkan AF Guclu-Geyik F Ekici B. Levels of miR-130b-5p in Peripheral Blood Are Associated With Severity of Coronary Artery Disease. Mol Biol Rep (2021) 48(12):7719–32. doi: 10.1007/s11033-021-06780-5 34689283

[35] Liu X Chen S Zhang L. Downregulated microRNA-130b-5p Prevents Lipid Accumulation and Insulin Resistance in a Murine Model of Nonalcoholic Fatty Liver Disease. Am J Physiol Endocrinol Metab (2020) 319(1):E34–42. doi: 10.1152/ajpendo.00528.2019 32228319

[36] Wang LP Gao YZ Song B Yu G Chen H Zhang ZW. MicroRNAs in the Progress of Diabetic Nephropathy: A Systematic Review and Meta-Analysis. Evid Based Complement Alternat Med (2019) 2019:3513179. doi: 10.1155/2019/3513179 30984273 PMC6431481

[37] Zhuang L Wang Z Hu X Yang Q Pei X Jin G. CircHIPK3 Alleviates High Glucose Toxicity to Human Renal Tubular Epithelial HK-2 Cells Through Regulation of miR-326/miR-487a-3p/SIRT1. Diabetes Metab Syndr Obes (2021) 14:729–40. doi: 10.2147/DMSO.S289624 PMC7898210 33628038

[38] Wang M Wei J Shang F Zang K Zhang P. Down-Regulation of lncRNA SNHG5 Relieves Sepsis-Induced Acute Kidney Injury by Regulating the miR-374a-3p/TLR4/NF-κb Pathway. J Biochem (2021) 169(5):575–83. doi: 10.1093/jb/mvab008 33479745

[39] Xu J Zhang P Sun H Liu Y. LINC01094/miR-577 Axis Regulates the Progression of Ovarian Cancer. J Ovarian Res (2020) 13(1):122. doi: 10.1186/s13048-020-00721-9 33069244 PMC7568364

[40] Yu P Wei K Zhang T Tan Z Zhao H Sun H. CircTMCO3 Promotes Gastric Cancer Progression by Regulating miR-577/RAB14 Axis. Cancer Manag Res (2021) 13:6079–88. doi: 10.2147/CMAR.S300559 PMC8349533 34377026

[41] Tang J Gao W Liu G Sheng W Zhou J Dong Q. miR-944 Suppresses EGF-Induced EMT in Colorectal Cancer Cells by Directly Targeting Gata6. Onco Targets Ther (2021) 14:2311–25. doi: 10.2147/OTT.S290567 PMC8020141 33833529

[42] Chen XY Li GM Dong Q Peng H. MiR-577 Inhibits Pancreatic β-Cell Function and Survival by Targeting Fibroblast Growth Factor 21 (FGF-21) in Pediatric Diabetes. Genet Mol Res (2015) 14(4):15462–70. doi: 10.4238/2015.November.30.24 26634512

[43] Parker DC Wan M Lohman K Hou L Nguyen AT Ding J. Monocyte miRNAs Are Associated With Type 2 Diabetes. Diabetes (2022) 71(4):853–61. doi: 10.2337/db21-0704 PMC8965663 35073575

