# Supplementary material for: Bioinformatics Analysis of the Mechanisms of Diabetic Nephropathy via Novel Biomarkers and Competing Endogenous RNA Network
Source: Front Endocrinol (Lausanne). 2022 Jul 14;13:934022. doi: 10.3389/fendo.2022.934022 (PMC9329782; doi:10.3389/fendo.2022.934022)
Supplement: Supplementary file 1 [file DataSheet_1.pdf]

The primer sequences.

Gene	Sense Primers (5’-3’)	Anti-sense Primers (5’-3’)
PTPRC	TTGAGCGACAGGAGGATGAG	GACGCCTCTCCACATTGCT
CD53	ATGATGGCAGGGTCCTTCATG	AGCTTCCTCAGCTAATGATTG
IRF8	CCAGCCAGTTCTTCCGA	CCTCTTCTGCCAGTTGCC
IL10RA	CACCCTGGATCTGTATCACCG	TAGATGATGCCGTCCATTGCT
LAPTM5	CGTCTCGTCTCCATCAGCAG	TGACCCATCCTGTCGTCTGA
GAPDH	CCTCGTCCCGTAGACAAAATG	TGAGGTCAATGAAGGGGTCGT
