# Supplementary material for: Bioinformatics Analysis of the Mechanisms of Diabetic Nephropathy via Novel Biomarkers and Competing Endogenous RNA Network
Source: Front Endocrinol (Lausanne). 2022 Jul 14;13:934022. doi: 10.3389/fendo.2022.934022 (PMC9329782; doi:10.3389/fendo.2022.934022)
Supplement: Supplementary file 2 [file DataSheet_2.pdf]

GSE30122

C1QA	Up
SERPINE2	Up
C1QB	Up
TMPRSS4	Up
LCK	Up
CD163	Up
RNASE6	Up
OLFML3	Up
CD1C	Up
SERPINA3	Up
VSIG4	Up
FN1	Up
THBS2	Up
CPA3	Up
MS4A6A	Up
MOXD1	Up
NNMT	Up
C7	Up
BZW2	Up
COL1A2	Up
COL6A3	Up
CD48	Up
CD53	Up
MARCKS	Up
LUM	Up
CD3D	Up
ADH1B	Up
MS4A4A	Up
TDO2	Up
KCNJ8	Up
RARRES1	Up
CDC20	Up
NELL2	Up
DHCR24	Up
GCNT3	Up
LTF	Up
VTCN1	Up
PLAAT4	Up
ITGB2	Up
P2RY14	Up
EVI2A	Up
ARL4C	Up
CD2	Up
C3	Up
LY86	Up
TLR1	Up
IRF8	Up
HLA-DPA1	Up

SPP1	Up
CST6	Up
CD52	Up
TYROBP	Up
BLNK	Up
ACKR1	Up
GZMA	Up
CORO1A	Up
SFN	Up
AP1M2	Up
GZMK	Up
GPX1	Up
CXCL6	Up
LAPTM5	Up
SERPINF1	Up
CCL19	Up
IL10RA	Up
WFDC2	Up
CAPG	Up
GPR171	Up
IL7R	Up
CCL5	Up
FCER1A	Up
PYCARD	Up
GABRP	Up
MRC1	Up
TGFBI	Up
MMP7	Up
SFRP1	Up
RRM2	Up
JCHAIN	Up
LTB	Up
GPR18	Up
CYP24A1	Up
TSPAN1	Up
HMOX1	Up
ABCA8	Up
PFN1	Up
CLU	Up
FMO3	Up
TLR7	Up
AGR2	Up
EVI2B	Up
RASGRP1	Up
LYZ	Up
TKT	Up
OGDHL	Up
OLFM4	Up
TNFAIP8	Up

AKR1B10	Up
PLAC8	Up
CRIP1	Up
CAPN3	Up
HOPX	Up
CHL1	Up
HABP2	Up
REG1A	Up
HLA-DRA	Up
SLC6A8	Up
SOX9	Up
GPR183	Up
UBD	Up
RPL13	Up
PTPRC	Up
TAGLN	Up
ALDH1A1	Up
SPINK1	Up
TAC1	Up
LY96	Up
PROM1	Up
S100A2	Up
KRT19	Up
ARHGAP19	Down
NPHS1	Down
PLCE1	Down
FYN	Down
TGFBR3	Down
SGCE	Down
CA10	Down
LOX	Down
P3H2	Down
TRAM2	Down
ZNF185	Down
CORO2B	Down
TCF7L1	Down
RIPOR1	Down
MAGI2	Down
PRKAR2B	Down
DACH1	Down
TJP1	Down
PARVA	Down
MPP5	Down
ZDHHC6	Down
DPP6	Down
NRIP2	Down
SEMA5A	Down
FERMT2	Down
DPYSL3	Down

B3GALT2	Down
LGALS8	Down
LRRC2	Down
CERS6	Down
KBTBD11	Down
ZNF415	Down
F5	Down
B4GAT1	Down
C1ORF21	Down
ARHGEF3	Down
RABL3	Down
CHI3L1	Down
SRGAP2	Down
TCEAL9	Down
TACC2	Down
FBXL7	Down
ANXA2	Down
SLC31A2	Down
CCDC91	Down
SULF1	Down
TNS3	Down
SYNPO	Down
POGLUT1	Down
KLK6	Down
THSD7A	Down
C2ORF68	Down
TCF21	Down
USP46	Down
NEBL	Down
GTF2A2	Down
BCAR3	Down
ARHGEF12	Down
ANKRD28	Down
MME	Down
CDC42EP3	Down
CTNNBIP1	Down
CDKN1C	Down
TNNT2	Down
BTG3	Down
TSPYL4	Down
SEMA3G	Down
CITED2	Down
PMEPA1	Down
NEK7	Down
KLF7	Down
VEGFA	Down
CXADR	Down
ARMCX1	Down
NES	Down

ST3GAL6	Down
CCSER2	Down
STON1	Down
RETREG1	Down
FRY	Down
SPOCK1	Down
MXRA8	Down
MAFB	Down
ZNF211	Down
GHR	Down
ITGA3	Down
SPOCK2	Down
C8ORF33	Down
DCN	Down
CD200	Down
ARRB1	Down
MXRA7	Down
NDN	Down
NECTIN2	Down
GPRC5A	Down
IQGAP2	Down
RASL11B	Down
PDPN	Down
CBX7	Down
AMIGO2	Down
STXBP1	Down
FAM114A1	Down
SYBU	Down
TNS2	Down
PTGDS	Down
ZNF274	Down
HTRA1	Down
F2R	Down
NDNF	Down
FOXD1	Down
PLA2R1	Down
ZFPM2	Down
GAS1	Down
BMP7	Down
PCOLCE2	Down
TSPYL5	Down
NFE2L3	Down
ZNF91	Down
ETS2	Down
PDLIM2	Down
FAM98A	Down
FGF9	Down
CETN3	Down
WT1	Down

FANCL	Down
SEPTIN11	Down
KLHL3	Down
MYOZ2	Down
FGF1	Down
KAT2B	Down
MGAT5	Down
ZNF423	Down
FOXC1	Down
EXPH5	Down
GJA1	Down
F3	Down
TPPP3	Down
NTNG1	Down
ELOVL4	Down
CLIP3	Down
AIF1	Down
EFEMP1	Down
FZD2	Down
MCM6	Down
DHRS3	Down
MYO1E	Down
NFASC	Down
SHROOM2	Down
JAM3	Down
KLK7	Down
NAP1L2	Down
LPL	Down
CLDN5	Down
PEA15	Down
UGGT2	Down
CPEB1	Down
BST1	Down
LHFPL6	Down
CREB3L2	Down
COLGALT2	Down
RAI14	Down
NR3C2	Down
ZNF804A	Down
CAND2	Down
TRIB2	Down
ACTN4	Down
MYLIP	Down
ITGB5	Down
UBAP2	Down
TSPAN5	Down
MYH9	Down
COL4A5	Down
GPNMB	Down

EMP2	Down
HPS5	Down
NEDD9	Down
TSPAN2	Down
MYO5C	Down
SLK	Down
TCEAL2	Down
ATP10A	Down
ANKMY2	Down
MYL9	Down
CLIC5	Down
HIVEP2	Down
NXN	Down
CCN2	Down
RASGRP3	Down
LIMS2	Down
MYLK3	Down
PALLD	Down
BEX4	Down
HS3ST3A1	Down
MANSC1	Down
FAM13B	Down
TMOD1	Down
CR1	Down
SOBP	Down
NAP1L3	Down
PTPRO	Down
NLK	Down
TNNI1	Down
OLFML2A	Down
ITIH5	Down
SNCA	Down
PHACTR4	Down
APOLD1	Down
MYOZ1	Down
C14ORF132	Down
NME7	Down
STN1	Down
WWC3	Down
BMP2	Down
WFS1	Down
TPM2	Down
PTPRD	Down
SERINC5	Down
CSRP1	Down
EMCN	Down
BCAM	Down
ZNF124	Down
ARHGAP28	Down

HPGD	Down
ZBTB10	Down
ZBED8	Down
DDN	Down
PODXL	Down
IFIT1	Down
PPFIA4	Down
GOLIM4	Down
NPHS2	Down
HMBOX1	Down
GADD45B	Down
GADD45A	Down
RNF220	Down
RBMS1	Down
HRG	Down
RRAS	Down
APOD	Down
EPB41L5	Down
ROBO1	Down
SERINC1	Down
HSPA2	Down
WASF3	Down
PCMTD2	Down
MED4	Down
ZNF302	Down
NQO1	Down
ZC2HC1A	Down
PHYHIP	Down
TNNC1	Down
EHD3	Down
PLBD1	Down
KANK3	Down
IL13RA2	Down
GMDS	Down
MYLK	Down
PSD3	Down
NXF3	Down
CTSV	Down
PAMR1	Down
SLC14A1	Down
PNMA8A	Down
CHN1	Down
NOTCH2NLA	Down
ZBTB16	Down
FBXO3	Down
PRSS16	Down
CFHR1	Down
SBSPON	Down
MAP1B	Down

XYLT1	Down
SOWAHC	Down
TMEM45A	Down
CDH10	Down
PLCG2	Down
CCN6	Down
EML1	Down
MTHFD2	Down
DYNC1I1	Down
CRHBP	Down
ETNPPL	Down
PLAT	Down
RAMP3	Down
EFNB2	Down
TYRP1	Down
CETP	Down
SST	Down
HECA	Down
ITGA8	Down
TIPARP	Down
IGF1	Down
PLTP	Down
PLPP3	Down
ERGIC2	Down
ITGBL1	Down
VILL	Down
RGS2	Down
TGFB1I1	Down
VIM	Down
SLC2A10	Down
IGFBP1	Down
CTSO	Down
RAPGEF4	Down
SGK1	Down
PTPRB	Down
KDR	Down
KIRREL1	Down
BAMBI	Down
RIDA	Down
NELL1	Down
EGF	Down
ID3	Down
IGFBP2	Down
MEIS2	Down
CDH13	Down
LDB2	Down
UMOD	Down
CFAP45	Down
FOSB	Down

APOH	Down
AFM	Down
TMSB15A	Down
CYP26B1	Down
