# Supplementary material for: Bioinformatics Analysis of the Mechanisms of Diabetic Nephropathy via Novel Biomarkers and Competing Endogenous RNA Network
Source: Front Endocrinol (Lausanne). 2022 Jul 14;13:934022. doi: 10.3389/fendo.2022.934022 (PMC9329782; doi:10.3389/fendo.2022.934022)
Supplement: Supplementary file 3 [file DataSheet_3.pdf]

GSE30529

CXCL6	Up
CASP1	Up
LYZ	Up
QPCT	Up
MMP7	Up
JCHAIN	Up
PLK2	Up
PTPRE	Up
EVI2B	Up
TRIM22	Up
COL4A1	Up
TNFAIP8	Up
MS4A4A	Up
C3	Up
SOD2	Up
POGLUT2	Up
NMI	Up
PDLIM1	Up
PTPRC	Up
VCAN	Up
CTSS	Up
HDAC9	Up
COL3A1	Up
AGR2	Up
PXDN	Up
LAPTM5	Up
HLA-DQA1	Up
CD48	Up
CD53	Up
PROM1	Up
C1S	Up
NNMT	Up
LTF	Up
MNDA	Up
EVI2A	Up
SPARC	Up
CSF2RB	Up
B2M	Up
CCR2	Up
FSTL1	Up
CPA3	Up
TPM1	Up
FCER1A	Up
JAK1	Up
PDIA6	Up
PSMB9	Up
ANXA3	Up
HLA-DPA1	Up

ITGB2	Up
CX3CR1	Up
CLU	Up
MID1	Up
PSMB8	Up
TAC1	Up
MARCKSL1	Up
NLGN4X	Up
PLSCR1	Up
CASP3	Up
PON2	Up
RCN1	Up
ANXA2	Up
COL15A1	Up
HLA-B	Up
HLA-DRA	Up
CCL2	Up
CFHR1	Up
CD163	Up
C1RL	Up
ANXA1	Up
TMEM45A	Up
VIM	Up
ECT2	Up
TNC	Up
HCLS1	Up
HLA-DMA	Up
IFI16	Up
LYN	Up
IRF8	Up
ARL4C	Up
PRKX	Up
CD1C	Up
HLA-DRB1	Up
ASNS	Up
GZMA	Up
TAX1BP3	Up
ITM2C	Up
COL1A2	Up
EFNB2	Up
TUBB	Up
SCRN1	Up
FZD7	Up
RNASE6	Up
ALOX5	Up
TLR7	Up
MNS1	Up
SLC2A10	Up
FCGR3B	Up

HLA-C	Up
HLA-DPB1	Up
HLA-DRB5	Up
SELL	Up
KLRB1	Up
MS4A6A	Up
MARCKS	Up
UBD	Up
TRIM16	Up
MAGED4B	Up
TIMP1	Up
LY75	Up
IRF9	Up
YWHAH	Up
COL6A3	Up
RRM2	Up
RARRES1	Up
MELK	Up
LUM	Up
CDH6	Up
PSTPIP2	Up
DSE	Up
HLA-F	Up
HLA-E	Up
LOXL1	Up
C1QB	Up
PRC1	Up
STK10	Up
IMPDH2	Up
CLEC2B	Up
RAB31	Up
ESYT1	Up
FN1	Up
WFDC2	Up
DHRS9	Up
RHOA	Up
PLAC8	Up
MRC1	Up
MYC	Up
CEP170	Up
VCAM1	Up
GNL3	Up
ZNF264	Up
TXNDC5	Up
CFH	Up
HTR2B	Up
BIRC3	Up
PLA2G4A	Up
TUBA1A	Up

FCGR2A	Up
SPRY1	Up
APOBEC3B	Up
ANXA5	Up
SOX4	Up
IGSF6	Up
ITGAV	Up
VSIG4	Up
CKLF	Up
CLEC4A	Up
SRGN	Up
KRT19	Up
TNFAIP6	Up
CHST15	Up
ARPC3	Up
TPBG	Up
NIBAN1	Up
SH2B3	Up
CORO1A	Up
DCK	Up
NEU1	Up
SPON2	Up
PLEKHA1	Up
SERPINA3	Up
TFPI2	Up
TUBA1B	Up
LY96	Up
PRKCB	Up
FABP5	Up
LY86	Up
TES	Up
REG1A	Up
SERPING1	Up
TGFBI	Up
TXNIP	Up
MOXD1	Up
TMSB4X	Up
CD2	Up
FCHSD2	Up
ACSL4	Up
IFI44L	Up
POU2AF1	Up
CRISPLD2	Up
BLNK	Up
KCTD12	Up
CLEC7A	Up
GABRP	Up
LPCAT1	Up
CCNB1	Up

PPIB	Up
BRD7	Up
CXCR4	Up
RAI2	Up
TMSB10	Up
IFNGR1	Up
TMEM243	Up
DOCK2	Up
CCL5	Up
NXN	Up
CSTB	Up
ACTN1	Up
FGL2	Up
MSL1	Up
FCGR2B	Up
ARPC1B	Up
HOPX	Up
ARHGDIB	Up
VOPP1	Up
CSTA	Up
GBP2	Up
IL10RA	Up
PFKP	Up
THBS2	Up
PFN1	Up
P2RY13	Up
MCUB	Up
ZEB2	Up
THYN1	Up
GCNT3	Up
TLR1	Up
CXCL1	Up
FCN1	Up
GPR18	Up
MAFB	Up
TSPAN13	Up
TYROBP	Up
MX1	Up
GLIPR1	Up
NFKBIE	Up
TDO2	Up
LHFPL6	Up
ARAP2	Up
SLC26A2	Up
SERPINE2	Up
SAE1	Up
GUCY1A1	Up
ACKR4	Up
ZNF652	Up

ENAH	Up
ADCY7	Up
PLP2	Up
DDX60	Up
PYCARD	Up
COL4A2	Up
MYO1D	Up
BHLHE41	Up
CFB	Up
MPHOSPH8	Up
SRPX	Up
LCP2	Up
MEX3C	Up
C7	Up
DACT1	Up
KRT8	Up
RPS3	Up
RPAP3	Up
CD74	Up
CD69	Up
PLAAT4	Up
LPGAT1	Up
ARHGAP15	Up
FHL2	Up
GPNMB	Up
CXCL12	Up
TUSC3	Up
DOK5	Up
NAA15	Up
ALDH1A3	Up
LTB	Up
RABL2B	Up
ACKR1	Up
POSTN	Up
CARMIL1	Up
CCL19	Up
FKBP11	Up
PSMB10	Up
WNT5A	Up
FMO3	Up
NCF2	Up
TUBA1C	Up
TSPAN1	Up
RACK1	Up
C1QA	Up
ARHGAP29	Up
S100A4	Up
VLDLR	Up
RND3	Up

CAPG	Up
AEBP1	Up
P2RY14	Up
CD9	Up
LAMP3	Up
IFITM2	Up
CXADR	Up
NR1D2	Up
IFITM3	Up
ISG20	Up
MTHFD2	Up
FCER1G	Up
OXR1	Up
GZMK	Up
CLEC10A	Up
ILF2	Up
FZD2	Up
C1R	Up
SAMSN1	Up
RTN3	Up
SLPI	Up
CRIP1	Up
IL7R	Up
TNFRSF17	Up
SNF8	Up
AKAP12	Up
VWF	Up
SEL1L3	Up
CST6	Up
CD3D	Up
A2M	Up
HLA-DMB	Up
RASSF2	Up
FLRT3	Up
HLA-DQB1	Up
COMP	Up
IFITM1	Up
PLTP	Up
IGFBP6	Up
SMARCD3	Up
CCL20	Up
IFI35	Up
VTCN1	Up
GMFG	Up
MGP	Up
BST2	Up
GPX1	Up
RPL8	Up
ZKSCAN7	Up

SFRP1	Up
TNMD	Up
HSP90AB1	Up
CYTIP	Up
HSPB1	Up
TRMT2B	Up
LCN2	Up
FGF13	Up
CD52	Up
ERAP2	Up
IGFBP2	Up
IFI27	Up
CXCL9	Up
CTSK	Up
SELENOT	Up
SLC44A4	Up
CYP24A1	Up
ALDH1A1	Up
HABP2	Up
IGLL5	Up
SERPINF1	Up
KRT18	Up
PRELID3B	Up
ADH1B	Up
FABP4	Up
BASP1	Up
TAGLN	Up
LGALS1	Up
SOX9	Up
ADAMTS1	Up
HRG	Down
PER1	Down
NELL1	Down
AVPI1	Down
EGF	Down
CGA	Down
PXMP2	Down
KLK1	Down
ASB9	Down
DNMT3L	Down
NR4A3	Down
STBD1	Down
LPL	Down
KLHL21	Down
USP2	Down
ARSF	Down
CEL	Down
LHPP	Down
BPI	Down

CYP3A7	Down
CLDN8	Down
METTL1	Down
GRIP2	Down
TYRP1	Down
OPCML	Down
THY1	Down
WNT10B	Down
CES3	Down
DEFB1	Down
CYP46A1	Down
FAM184A	Down
SLC2A4RG	Down
CYP4F12	Down
TCF15	Down
FMO5	Down
PROC	Down
KNG1	Down
SORD	Down
RAB11FIP3	Down
GADD45B	Down
SLC46A3	Down
STRA6	Down
APOH	Down
CYP27B1	Down
RBP4	Down
DIP2C	Down
SLC4A1	Down
UPB1	Down
GADD45G	Down
TSKU	Down
ACSM5	Down
KCNN2	Down
RALYL	Down
APOC3	Down
CIDEB	Down
NR1I3	Down
RNF186	Down
SLC39A4	Down
CD83	Down
FGF9	Down
SPATA2L	Down
ETNPPL	Down
TCEAL2	Down
FTCD	Down
EAF2	Down
AOC1	Down
GHR	Down
PPP1R16B	Down

UMOD	Down
ZGPAT	Down
SERPINA6	Down
KL	Down
PVALB	Down
PIPOX	Down
FOSB	Down
DCXR	Down
GLYAT	Down
ACP5	Down
COX7A1	Down
ACKR3	Down
SCGB1D2	Down
MYH8	Down
ASPA	Down
ALDOB	Down
HAO2	Down
FBP1	Down
DAO	Down
ADIRF	Down
GPC5	Down
APOM	Down
DPEP1	Down
LEFTY1	Down
GC	Down
TDGF1	Down
ANGPTL3	Down
ALB	Down
MME	Down
DIO1	Down
